# The association between state-level negative racial sentiment and maternal hypertension in the US from 2016 to 2021: An observational study using Twitter data

**DOI:** 10.1371/journal.pone.0346564

**Published:** 2026-04-29

**Authors:** Laura B. Drew, Junaid Merchant, Marie E. Thoma, Heran Mane, Xiaohe Yue, Thu T. Nguyen

**Affiliations:** 1 Department of Epidemiology and Biostatistics, Michigan State University, East Lansing, Michigan, United States of America; 2 Department of Epidemiology and Biostatistics, School of Public Health, University of Maryland, College Park, Maryland, United States of America; 3 Department of Family Science, School of Public Health, University of Maryland, College Park, Maryland, United States of America; Korea University - Seoul Campus: Korea University, KOREA, REPUBLIC OF

## Abstract

**Background:**

Racial disparities in maternal hypertension (i.e., prepregnancy and gestational) due to experiences of racism may contribute to ongoing racial disparities in US birth outcomes, including low birth weight and preterm birth. Despite evidence linking racism and adverse birth outcomes, no studies have examined the plausible association between area-level negative racial sentiment and disparities in maternal hypertension. To address this gap, we used 2016–2021 US birth certificate data to examine the associations between state-level Twitter-derived negative sentiments toward racial/ethnic minorities and maternal hypertension. We further examined if these associations increased during periods of heightened racial discrimination.

**Methods:**

We used 2016–2021 US natality data with geographic identifiers for pregnancy data for singleton births (n = 22,618,566) and a random sample of 1% of publicly available tweets from 2016–2021 (n = 56,400,097) using Twitter’s Academic Application Programming Interface. We calculated annual state-level negative racial sentiment by averaging sentiment scores of all posts referencing a racial category. These scores, divided into quartiles, included five sentiment measures: one toward all racially minoritized groups and four race-specific (Black, Asian, Latinx, and White). We merged data for each year and used log-binomial regression to estimate prevalence rate ratios (PRRs) for prepregnancy and gestational hypertension, adjusting for individual maternal characteristics and state-level demographics. We additionally stratified analyses into time periods before and during the COVID-19 pandemic and Black Lives Matter movement (2016–2019 and 2020–2021, respectively).

**Results:**

In our sample, 2.2% of individuals had prepregnancy hypertension and 7.7% had gestational hypertension. From 2016−2021, the prevalence of both types of maternal hypertension increased for individuals across all racial and ethnic groups. Individuals in states with the highest quartile of negative racial minority sentiment had a 36% higher (95% CI: 1%−83%) prevalence of prepregnancy hypertension and a 20% higher (95% CI: 0%−45%) prevalence of gestational hypertension compared to those in the lowest quartile. In 2020 and 2021, the prevalence of prepregnancy hypertension among individuals in racially minoritized groups was 51% greater (95% CI:12%−103%) in the 4^th^ quartile compared to the 1^st^ quartile and showed a higher magnitude of association compared to 2016−2019. In 2020−2021, among Black individuals, those in the highest quartile of anti-Black sentiment had a 31% higher (95% CI: 2%−69%) prevalence of prepregnancy hypertension, and the 19% increase in prepregnancy hypertension among Asian individuals in the highest quartile of anti-Asian sentiment was borderline significant (95% CI: 0%−44%). Patterns for gestational hypertension were not consistent across time and when examining race-specific sentiment.

**Conclusion:**

Higher levels of state-level negative racial sentiment are associated with increased prevalence of prepregnancy and gestational hypertension among racially minoritized groups, and the association with prepregnancy hypertension appears to strengthen during periods of heightened racial tension and discrimination. These findings highlight the role of area-level racism as a contributor to maternal health disparities.

## Introduction

In the United States, Black women have higher rates of maternal morbidity and mortality as well as adverse birth outcomes, including low birth weight (LBW), preterm birth (PTB), and infant mortality compared to White women [1–8]. While most studies focus on risk and protective factors during pregnancy as the causes of these Black-White disparities, individual-level factors do not sufficiently explain why racial disparities in maternal health and birth outcomes persist [9]. Increasing evidence posits that racism is a root cause of racial health disparities in the U.S [9–11]. Hypertension preceding pregnancy (i.e., prepregnancy/chronic hypertension) and gestational hypertension are well-established risk factors for adverse birth outcomes, including low birth weight (LBW), very low birth weight, (VLBW), and preterm birth (PTB) [12,13]. Therefore, it is plausible that racial disparities in maternal hypertension—due to experiences of racism—may contribute to the persistent racial disparities in birth outcomes in the U.S.

Racism functions across many dimensions, including internalized, personally mediated (i.e., individual or interpersonal), and institutional/structural [14], and these types of racism can negatively impact one’s health. Internalized racism is when members of the stigmatized races accept the negative messages that they receive from others outside their race about their abilities and worth [14]. Personally mediated racism encompasses experiences of prejudice and discrimination enacted between individuals, both intentional and unintentional, and institutionalized/structural racism pertains to differential access to resources, such as education, safe housing, and medical facilities [14]. In addition to these three dimensions of racism, cultural racism can contribute to racial inequities in health [15]. Cultural racism is discriminatory cultural messaging that communicates and supports the belief that racial and ethnic groups have different statuses and rights, while systematically oppressing those who are non-White and reinforcing the belief in White cultural superiority [15]. Cultural racism is expressed through media, stereotyping, norms, and beliefs within society and its institutions [16], and it is strengthened through policies and practices within and across institutions that further sustain health inequities [17,18]. Collectively, these forms of racism maintain and underpin a hierarchical system of oppression and discrimination that contributes to racial disparities in the US [19]. From a life course perspective, addressing these overlapping forms of racism is critical; the cumulative impact of racial discrimination over time, including prior to pregnancy, can significantly affect maternal health, making preconception care and early intervention key strategies in reducing adverse birth outcomes [9].

Increasing evidence suggests racial bias and discrimination may contribute to the persistent disparities in LBW and PTB [10,11,20–22]. However, experiences with discrimination and racism are usually assessed at the individual level by self-reports, and these measures are subject to limitations including social desirability and self-censorship [23]. Additionally, they may not accurately account for the complex experience of racism [24]. Thus, more research is needed to investigate how experiences of other dimensions of racism can impact health and highlight areas that can be targeted for intervention. Measures of the social climate in particular locations via social media data, like Twitter (now known as X), have been shown as a proxy and/or complimentary measure for experiences of discrimination and racism in the U.S. at the state and county level [10,11,25–30]. Previous research assessing Twitter-derived negative racial sentiment found an increased incidence of LBW and PTB among Black and Latinx mothers who lived in states with high negative sentiment toward their respective races [11].

Our understanding of the vascular processes that mediate the relationship between social adversity experiences before and during pregnancy and adverse birth outcomes is still in development [31]. However, clinical studies have demonstrated that exposure to racial stressors can trigger physiological responses linked to hypertension, particularly among racial and ethnic minorities [32–34], and numerous lines of evidence indicate a relationship between racism and cardiovascular responses and hypertension [24,35–38]. Chronic stress during pregnancy can affect maternal cardiovascular, inflammatory, and neuroendocrine mechanisms, which can negatively impact infants’ birthweight and initiate preterm birth [31,39,40]. In addition, studies have shown socio-historical events such as police violence, the Black Lives Matter movement, and the COVID-19 pandemic, disproportionately increased stress and hypertension among racial minorities [41,42]. Similarly, adverse birth outcomes among non-white and immigrant mothers were observed following the 2016 US presidential election [43,44].

Despite the well-documented links between racism and adverse birth outcomes, no studies have examined the association between area-level negative racial sentiment and disparities in maternal hypertension (prepregnancy and gestational), which contributes to approximately 10% and 8% of the Black-White disparity in PTB and very preterm birth (VPTB), respectively [12]. To address this gap, this study utilizes 2016−2021 US birth certificate data to examine the associations between state-level Twitter-derived negative sentiments toward racial/ethnic minorities and maternal hypertension (i.e., prepregnancy and gestational hypertension). Our analyses further disaggregate this relationship by comparing the periods before (2016−2019) and during (2020−2021) the COVID-19 pandemic and Black Lives Matter demonstrations, which were periods of heightened racial discrimination toward Asian and Black communities [30,45–47].

## Methods

### Twitter data

A random sample of 1% of publicly available tweets from 2016–2021 was collected using Twitter’s Academic Application Programming Interface. Like Nguyen et al., [11] we restricted analyses to tweets that included a unique “tweet id,” were in English, originated from the United States, and used one or more race-related keywords, which were developed based on racial and ethnic terms from the US Census, prior studies that examined race-related online conversations [48], and a web-based database of racial slurs [49]. We removed duplicate tweets based on their “tweet_id,” but included retweets and quoted tweets as these account for nearly half of Twitter users’ posts [50]. The final analytic sample included 56,400,097 tweets referencing different racial categories from 3,699,646 users.

### Sentiment analysis

A support vector machine (SVM) (i.e., a supervised machine learning model) assessed the sentiment of each tweet. A full description of our model has been previously described [10,11]. The training data included 6481 manually labeled tweets by our research group and labeled tweets from Sentiment140 (n = 498), Kaggle (n = 7086), and Sanders (n = 5113), which are publicly available training datasets for sentiment analysis. Five-fold cross-validation was conducted to assess model performance, which achieved a high level of accuracy for negative sentiment classification (91%) and a high F1 score (84%). We measured accuracy as the number of posts with the correct prediction over the total number of tweets in the testing dataset. The F1 score measure balances positive predicted value and recall sensitivity (i.e., precision). Therefore, a high F1 score suggests the model is robust in predicting posts labeled as 1 (i.e., negative). Finally, we used the trained SVM model to identify tweets based on overall emotional tone, which could include an unfavorable opinion, hostile language, or prejudice (which could be positive or negative) by classifying each tweet that mentioned race for negative sentiment (i.e., binary classification; 1 = negative, 0 = not negative). Our state-level negative racial sentiment measures were calculated by averaging the sentiment scores of all the posts referencing a racial category posted within each state (per year), such that negative racial sentiment reflects the proportion of posts referencing a group that are classified as negative. We used state-level negative racial sentiment quartiles in our statistical models examining the association between maternal hypertension and negative racial sentiment. The code for data collection and sentiment analysis has previously been published [11,51]. We used five negative racial sentiment measures – one global measure of negative sentiment toward all racially minoritized groups and four race-specific negative sentiment measures toward individual racial/ethnic groups (Black, Asian, Latinx, and White).

### Individual-level pregnancy data

2016−2021 pregnancy data was obtained from US natality files (i.e., US Birth Certificate data files) with geographic identifiers. We selected this date range because all states began using the revised birth certificate in 2016, and we wanted to examine the relationship before and during the COVID-19 pandemic. We restricted analyses to singleton births due to the increased risk of hypertensive disorders among multifetal gestations [52,53]. The primary outcomes were prepregnancy and gestational hypertension. If neither prepregnancy hypertension nor gestational hypertension were checked, but eclampsia was checked, they were considered as having gestational hypertension. On the birth certificate data file, prepregnancy hypertension is defined as hypertension diagnosis prior to the onset of the current pregnancy, and does not include gestational/pregnancy-induced hypertension, while gestational hypertension is defined as hypertension diagnosis in the current pregnancy, including pregnancy-induced hypertension and preeclampsia, but the type of gestational hypertension is not specified [54]. Although the birth certificate data represents 100% of all registered live births in the US [55], hypertension is subject to underreporting [56,57].

### Demographic characteristics and covariates

Based on the literature, we examined variables that could confound the relationship between area-level racial sentiment and maternal hypertension. Individual-level demographic characteristics and potential covariates included maternal age (years), race (non-Hispanic White, non-Hispanic Black, Hispanic, non-Hispanic Asian, non-Hispanic Native Hawaiian or other Pacific Islander, non-Hispanic American Indian Alaskan Native, non-Hispanic Multiple Races/Unknown/Other), education (less than high school, high school, some college, bachelor’s degree or higher), nativity (US-born or born outside of US), marital status (married, unmarried with paternity acknowledgment, or unmarried without paternity acknowledgment), infant sex (male or female), parity (first born or second born/higher), BMI (underweight, normal, overweight, obese), timing of prenatal care initiation (first trimester, second trimester, and third trimester or none), smoking during pregnancy (yes, no), and complete smoking history (never, before pregnancy only, during pregnancy only, or both before and during pregnancy). We additionally adjusted for the following state-level characteristics, which were retrieved from the American Community Survey [58]: 1) proportion of non-Hispanic Black and Latinx individuals, 2) population density (per square mile), and 3) economic disadvantage (standardized factor score), which summarizes the state-level percentage of a number of characteristics (unemployed, some college education, high school diploma, children in poverty, single parent household, and median household income) to account for compositional differences in demographic and economic characteristics at the state level. The economic disadvantage factor score has been previously validated and used elsewhere [11,59].

### Statistical analyses

We merged the two data files (i.e., state-level negative racial sentiment data with birth data) by state and year and coded negative racial sentiment into quartiles to assess associations with birth outcomes. We calculated separate prevalence rate ratios (PRRs) for prepregnancy and gestational hypertension using log-binomial regression models. In our main models, the key independent variable was state-level negative racial sentiment, and we adjusted for individual maternal characteristics (age, race, nativity, and education) and state-level demographics (proportion of non-Hispanic Black and Latinx individuals, population density (per square mile), and economic disadvantage factor score). We conducted complete case analysis with <5% of the sample missing data on variables of interest and assessed statistical significance at p < 0.05. To examine how these associations might have varied over time, we stratified the analyses into time periods before and during the COVID-19 pandemic and Black Lives Matter movement (2016–2019 and 2020–2021, respectively). Our overall model investigated the relationship between state-level sentiment toward *all* racially minoritized groups and prepregnancy and gestational hypertension among the full sample, among pregnant people in racially minoritized groups, and White pregnant people separately. In our race-specific model, we individually explored the relationship between state-level negative sentiment toward specific racial groups (Black, Asian, Latinx, and White) and prepregnancy and gestational hypertension among pregnant people in each racial/ethnic group (Black, Asian, Latinx, and White).

To assess if additional factors influenced our findings, we conducted multiple sensitivity analyses. We stratified our main models by parity (primiparous and parous) and tested for an interaction with parity to assess if parity moderated the relationship between area-level racial sentiment and maternal hypertension. Because we could not differentiate the temporal relationship between other individual-level health risks and whether they operated as mediators or confounders in the association between racial sentiment and maternal hypertension, we further adjusted for these potential confounders separately: 1) BMI, 2) timing of prenatal care initiation, 3) smoking during pregnancy, and 4) complete smoking history. All statistical analyses were performed using Stata MP 18 [60]. We also conducted E-value analyses to assess the potential of unmeasured confounding [61]. The data collection and analysis methods for this study complied with the terms and conditions for the source of data.

### Ethics statement

This study was exempt upon review by the University of Maryland College Park Institutional Review Board (1797788−1).

## Results and discussion

**Fig 1:**
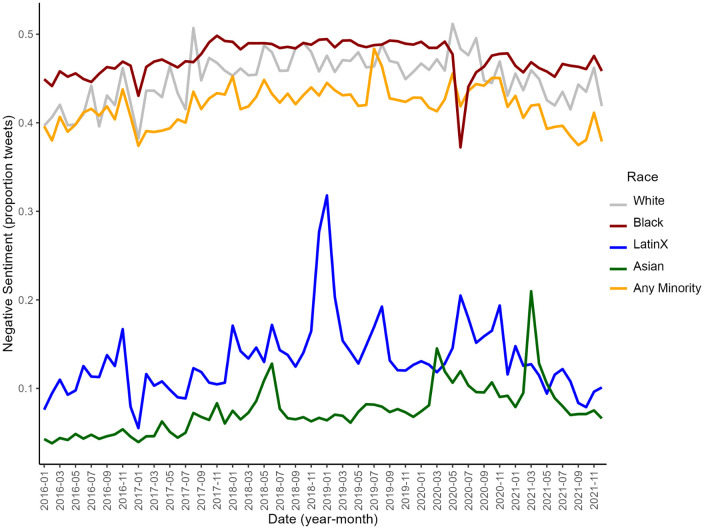
Temporal trends in the proportion of tweets from 2016-2020 that were negative toward all minority groups and individual racial and ethnic groups (White, Black, Latinx, and Asian).

**Fig 2:**
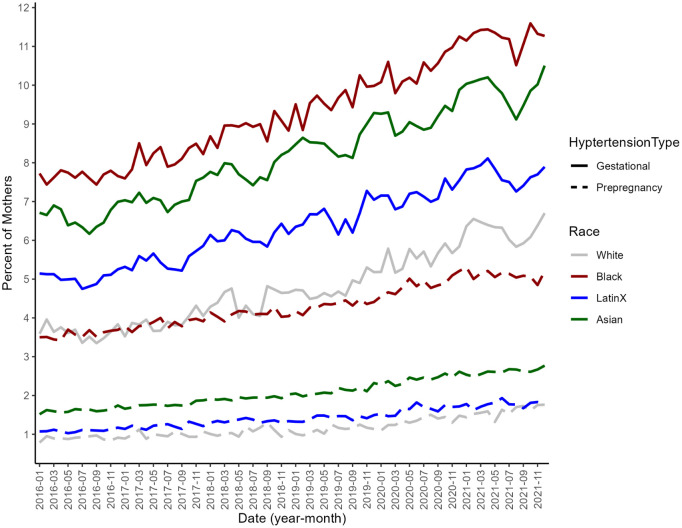
Temporal trends in prepregnancy and gestational hypertension from 2016-2021 among all pregnant mothers by racial and ethnic group (White, Black, Latinx, and Asian) based on US Birth Certificate Data.

In our sample (N = 22,618,566), 51.5% of births were to non-Hispanic White individuals, 23.6% were to Hispanic individuals, and 14.4% were to non-Hispanic Black individuals (Table 1). Most individuals were born in the US (77.5%), completed college or more (41.7%), had a second or higher-order birth (62.0%), and initiated prenatal care during the first trimester (77.6%). Overall, 2.2% of the sample had prepregnancy hypertension and 7.7% had gestational hypertension. From 2016–2021, the proportion of negative sentiment tweets toward all minority groups slightly increased and then decreased, overall resulting in a small change from 40.61% in 2016 to 39.93% in 2021 (Fig 1). Negative sentiment tweets toward Black individuals increased over time, but sharply decreased in May-July of 2020. The proportion of negative sentiment tweets referencing Asian individuals increased from 2016–2021 with peaks during March of 2020 and 2021. Prepregnancy and gestational hypertension increased among all pregnant people in every racial and ethnic group (White, Black, Latinx, and Asian) from 2016–2021 (Fig 2).

**Table 1:** Demographic characteristics, covariates, and outcomes of mothers giving birth from 2016-2021 (overall and by quartiles).

	State-Level Negative Minority Sentiment Quartiles				
	1	2	3	4	Overall
	N = 5,413,063	N = 3,962,907	N = 7,918,135	N = 5,324,461	N = 22,618,566
**Age** (mean, SD)	29.7 (5.8)	29.3 (5.8)	28.8 (5.8)	28.4 (5.7)	29.0 (5.8)
**Race** (%)	N = 5,413,063	N = 3,962,907	N = 7,918,135	N = 5,324,461	N = 22,618,566
NH White	43.5	56.3	50	58.4	51.5
NH Black	6.2	9.7	15.7	24.5	14.4
Hispanic	32.4	22.2	27	10.6	23.6
NH Asian	11	6	4.9	3.6	6.3
NH Native Hawaiian or other Pacific Islander	0.6	0.3	0.1	0.1	0.3
NH American Indian Alaskan Native	1.1	1.9	0.3	0.3	0.8
NH Multiple Races/Unknown/Other	5.2	3.6	1.9	2.5	3.1
**Nativity** (%)	N = 5,403,601	N = 3,956,915	N = 7,901,939	N = 5,310,390	N = 22,572,845
U.S. Born	71.2	77.6	76.4	85.5	77.5
Foreign Born	28.8	22.4	23.6	14.5	22.5
**Education** (%)	N = 5,225,282	N = 3,920,396	N = 7,864,071	N = 5,296,305	N = 22,306,054
Less than high school	12.2	12.3	12.7	12.7	12.5
High School	23.7	23.5	27.4	27.5	25.9
Some college	20.1	19.9	19.4	20.2	19.8
College or more	44.0	44.3	40.5	39.5	41.7
**Birth Order** (%)	N = 5,400,647	N = 3,950,420	N = 7,892,705	N = 5,309,771	N = 22,553,543
First	38.4	38.1	38.0	37.7	38.0
Second or more	61.6	61.9	62.0	62.3	62.0
**Prenatal Care Initiation** (%)	N = 5,305,101	N = 3,868,042	N = 7,686,479	N = 5,215,902	N = 22,075,524
1st Trimester	82.9	79.1	74.2	76.0	77.6
2nd Trimester	12.7	15.1	18.4	17.3	16.2
3rd Trimester or none	4.4	5.8	7.4	6.7	6.2
**Body Mass Index (BMI)** (%)	N = 5,283,078	N = 3,873,758	N = 7,736,325	N = 5,203,493	N = 22,096,654
Underweight	3.1	3.1	3.1	3.1	3.1
Normal	43.4	43.2	41.1	39.7	41.7
Overweight	27.0	26.7	27.0	26.1	26.7
Obese	26.5	27.0	28.8	31.1	28.5
**Complete Smoking History** (%)	N = 5,384,465	N = 3,946,830	N = 7,882,069	N = 5,295,273	N = 22,508,637
Never	94.8	91.8	92.2	88.8	92.0
Before pregnancy only	1.4	2.0	1.8	2.6	1.9
During pregnancy only	0.1	0.1	0.1	0.2	0.1
Before and during pregnancy	3.8	6.1	5.9	8.4	6.0
**Smoking During Pregnancy** (%)	N = 5,385,394	N = 3,947,369	N = 7,883,651	N = 5,296,574	N = 22,512,988
Yes	5.1	8.2	7.7	11.0	8.0
No	94.9	91.9	92.3	89.0	92.1
**Hypertension** (%)	N = 5,408,658	N = 3,958,495	N = 7,908,423	N = 5,320,064	N = 22,595,640
None	91.7	90.3	90.3	88.3	90.1
Prepregnancy	1.6	2.1	2.1	3	2.2
Gestational	6.7	7.7	7.6	8.7	7.7

N changes due to missing values.

From 2016−2021 and after adjustment for potential confounders, individuals from minoritized groups in states with higher negative racial minority sentiment (quartiles 2−4) (i.e., tweets referencing racial and ethnic minoritized groups) were generally associated with higher risk of prepregnancy and gestational hypertension compared to those in quartile 1. In particular, those from states with the highest (4^th^) quartile for negative racial minority sentiment had a 36% higher (95% CI: 1%−83%) prevalence of prepregnancy hypertension and a 20% higher (95% CI: 0%−45%) prevalence of gestational hypertension (Table 2). In 2020 and 2021, among racial minority groups, similar patterns held with all higher quartiles of negative racial minority sentiment associated with a significantly higher prevalence of prepregnancy hypertension among individuals in racially minoritized groups relative to quartile 1 and showed a higher magnitude of association compared with 2016−2019 data years. In particular, the prevalence of prepregnancy hypertension in 2020−2021 was 51% greater (95% CI: 12%−103%) among the 4th quartile compared with the 1st quartile, while the 4^th^ quartile of negative racial sentiment towards racial/ethnic minorities was only 1.23 times greater in 2016−2019 (95% CI: 0.88–1.73). From 2020−2021, there was an association between negative racial minority sentiment and maternal hypertension (prepregnancy and gestational) among White birthing people when comparing the highest quartile of negative racial sentiment to the 1st quartile (aPRR = 1.32, 95% CI:1.03–1.69 and aPRR = 1.18, 95% CI: 1.00–1.39, respectively) (Table 2).

**Table 2:** Unadjusted and adjusted associations using prevalence rate ratios (PRRs) between state-level negative racial sentiment toward minoritized groups and hypertension type by pregnant individual’s race (all, racially minoritized groups, White).

	All		Racially Minoritized Groups		White	
	Unadjusted PRR (95% CI)	Adjusted PRR (95% CI)	Unadjusted PRR (95% CI)	Adjusted PRR (95% CI)	Unadjusted PRR (95% CI)	Adjusted PRR (95% CI)
	2016-2021					
	N = 21,515,904		N = 10,354,781		N = 11,161,123	
**Prepregnancy**						
2^nd^ Quartile	1.30 (0.95-1.77)	1.12 (0.97-1.29)	1.52 (1.06-2.18)*	1.26 (1.05-1.51)*	1.11 (0.88-1.41)	1.03 (0.91-1.16)
3^rd^ Quartile	1.34 (0.94-1.90)	1.16 (0.97-1.38)	1.52 (1.06-2.18)*	1.24 (1.00-1.54)	1.21 (0.93-1.57)	1.12 (0.96-1.30)
4^th^ Quartile	1.92 (1.39-2.65)*	1.26 (0.97-1.64)	1.52 (1.06-2.18)*	1.36 (1.01-1.83)*	1.50 (1.17-1.92)*	1.20 (0.93-1.55)
**Gestational**						
2^nd^ Quartile	1.16 (0.93-1.45)	1.09 (0.96-1.22)	1.23 (0.99-1.52)	1.16 (1.00-1.35)	1.08 (0.90-1.29)	1.04 (0.94-1.15)
3^rd^ Quartile	1.16 (0.93-1.43)	1.10 (0.96-1.26)	1.19 (0.97-1.45)	1.17 (0.99-1.37)	1.10 (0.92-1.33)	1.07 (0.94-1.22)
4^th^ Quartile	1.36 (1.08-1.71)*	1.17 (1.00-1.36)	1.43 (1.14-1.79)*	1.20 (1.00-1.45)	1.26 (1.04-1.53)*	1.15 (0.97-1.35)
	2016-2019					
	N = 14,592,908		N = 7,009,713		N = 7,583,195	
**Prepregnancy**						
2^nd^ Quartile	1.31 (0.92-1.86)	1.10 (0.99-1.23)	1.55 (1.04-2.33)*	1.22 (1.06-1.41)*	1.11 (0.85-1.45)	1.03 (0.92-1.15)
3^rd^ Quartile	1.34 (0.92-1.95)	1.13 (0.94-1.34)	1.49 (0.93-2.38)	1.19 (0.96-1.48)	1.19 (0.90-1.57)	1.09 (0.92-1.29)
4^th^ Quartile	1.95 (1.26-2.79)*	1.16 (0.85-1.57)	2.58 (1.69-3.95)*	1.23 (0.88-1.73)	1.50 (1.13-1.98)*	1.12 (0.82-1.52)
**Gestational**						
2^nd^ Quartile	1.15 (0.88-1.51)	1.08 (0.95-1.23)	1.23 (0.96-1.59)	1.17 (0.99-1.39)	1.06 (0.84-1.32)	1.04 (0.93-1.16)
3^rd^ Quartile	1.16 (0.90-1.50)	1.11 (0.97-1.28)	1.23 (0.97-1.55)	1.20 (1.00-1.44)	1.09 (0.88-1.35)	1.07 (0.93-1.22)
4^th^ Quartile	1.38 (1.06-1.8)*	1.17 (0.99-1.38)	1.47 (1.13-1.91)*	1.24 (0.97-1.57)	1.26 (1.01-1.51)*	1.13 (0.96-1.34)
	2020-2021					
	N = 6,922,996		N = 3,345,068		N = 3,577,928	
**Prepregnancy**						
2^nd^ Quartile	1.26 (0.96-1.66)	1.13 (0.93-1.38)	1.45 (1.07-1.97)*	1.30 (1.04-1.63)*	1.11 (0.89-1.38)	1.02 (0.85-1.22)
3^rd^ Quartile	1.30 (0.94-1.79)	1.20 (0.98-1.48)	1.36 (0.92-2.02)	1.29 (1.02-1.62)*	1.22 (0.94-1.58)	1.17 (0.98-1.40)
4^th^ Quartile	1.83 (1.38-2.42)*	1.39 (1.06-1.81)*	2.31 (1.65-3.22)*	1.51 (1.12-2.03)*	1.47 (1.17-1.85)*	1.32 (1.03-1.69)*
**Gestational**						
2^nd^ Quartile	1.17 (1.01-1.37)*	1.09 (0.98-1.22)	1.22 (1.06-1.41)*	1.15 (1.10-1.31)*	1.11 (0.97-1.26)	1.05 (0.96-1.16)
3^rd^ Quartile	1.12 (0.95-1.33)	1.08 (0.95-1.23)	1.11 (0.94-1.30)	1.11 (0.85-1.30)	1.12 (0.96-1.30)	1.07 (0.95-1.22)
4^th^ Quartile	1.30 (1.09-1.55)*	1.17 (1.00-1.37)	1.34 (1.12-1.60)*	1.17 (0.98-1.38)	1.24 (1.05-1.46)*	1.18 (1.00-1.39)

1^st^ Quartile is the reference. Models adjusted for maternal characteristics (age, race, and education) and state-level demographic factors.

From 2016−2021, negative Black and Asian sentiments were associated with a significantly higher prevalence of prepregnancy hypertension among Black and Asian individuals in the highest quartile (q4) compared to the lowest quartile (Table 3). After stratifying into two time periods, this was not significant for Asian individuals from 2016−2019, but remained borderline significant in 2020−2021 with a higher magnitude of association (aPRR = 1.19, 95% CI: 0.99–1.44). Black individuals in the highest quartile of negative Black sentiment experienced a 31% (95% CI 2%−69%) higher prevalence in prepregnancy hypertension in 2020−2021. Other racial minority groups did not demonstrate consistent patterns or statistically significant associations with gestational hypertension, but gestational hypertension was higher among White individuals in the 4^th^ quartile of negative white sentiment in 2020−2021 (aPRR = 1.20, 95% CI: 1.02–1.20), compared to the lowest quartile. Sensitivity analyses did not result in significant changes to the models (Supplemental Tables 1 and 2 in S1 and S2 Tables), but did suggest the observed associations could be susceptible to modest, unmeasured confounding (S3 Table).

**Table 3:** Adjusted associations using prevalence rate ratios (PRRs) between state-level negative sentiment toward specific racial groups (Asian, Black, Latinx, and White) and hypertension type by pregnant individual’s race (Black, Asian, Latinx, White).

	Negative Black sentiment, Hypertension(s) among Black Mothers Adjusted PRR (95% CI)	Negative Asian sentiment, Hypertension(s) among Asian Mothers Adjusted PRR (95% CI)	Negative Latinx sentiment, Hypertension(s) among Latinx Mothers Adjusted PRR (95% CI)	Negative White sentiment, Hypertension(s) among White Mothers Adjusted PRR (95% CI)
	2016-2021			
	N = 3,091, 252	N = 1,351,154	N = 5,127,775	N = 11,161,123
**Prepregnancy**				
2^nd^ Quartile	1.26 (1.08-1.46)*	1.0 (0.92-1.09)	0.92 (0.84-1.00)	0.98 (0.86-1.12)
3^rd^ Quartile	1.21 (0.96-1.54)	1.01 (0.9-1.12)	0.98 (0.87-1.09)	1.03 (0.88-1.2)
4^th^ Quartile	1.39 (1.05-1.84)*	1.14 (1.01-1.28)*	1.12 (0.97-1.30)	0.97 (0.84-1.1)
**Gestational**				
2^nd^ Quartile	1.10 (1.00-1.20)	0.95 (0.85-1.07)	0.99 (0.92-1.07)	1.03 (0.97-1.11)
3^rd^ Quartile	1.09 (0.93-1.28)	0.92 (0.83-1.02)	1.02 (0.90-1.16)	1.07 (0.98-1.16)
4^th^ Quartile	1.16 (0.97-1.39)	0.95 (0.81-1.12)	1.06 (0.92-1.22)	1.07 (0.99-1.15)
	2016-2019			
	N = 2,099,548	N = 937,087	N = 3,447,687	N = 7,583,195
**Prepregnancy**				
2^nd^ Quartile	1.26 (1.04-1.53)*	0.99 (0.91-1.09)	0.95 (0.86-1.05)	0.93 (0.81-1.07)
3^rd^ Quartile	1.24 (0.93-1.66)	0.95 (0.83-1.09)	1.07 (0.96-1.18)	1.00 (0.85-1.18)
4^th^ Quartile	1.41 (0.99-2.01)	1.10 (0.97-1.26)	1.15 (0.94-1.4)	0.95 (0.84-1.08)
**Gestational**				
2^nd^ Quartile	1.11 (0.99-1.26)	0.98 (0.90-1.06)	1.02 (0.93-1.11)	1.08 (0.98-1.19)
3^rd^ Quartile	1.12 (0.94-1.33)	0.91 (0.82-1.01)	1.04 (0.92-1.17)	1.10 (0.98-1.22)
4^th^ Quartile	1.18 (0.97-1.45)	0.95 (0.80-1.13)	1.06 (0.92-1.21)	1.06 (0.96-1.18)
	2020-2021			
	N = 991,704	N = 414,067	N = 1,680,088	N = 3,577,928
**Prepregnancy**				
2^nd^ Quartile	1.22 (0.99-1.51)	0.99 (0.86-1.13)	0.79 (0.62-1.00)	1.05 (0.86-1.29)
3^rd^ Quartile	1.15 (0.91-1.44)	1.07 (0.93-1.22)	0.83 (0.69-1.00)	1.07 (0.88-1.29)
4^th^ Quartile	1.31 (1.02-1.69)*	1.19 (0.99-1.44)	1.02 (0.87-1.20)	0.99 (0.79-1.25)
**Gestational**				
2^nd^ Quartile	1.06 (0.94-1.19)	0.86 (0.7-1.05)	0.89 (0.78-1.0)	0.99 (0.90-1.09)
3^rd^ Quartile	1.04 (0.88-1.23)	0.90 (0.72-1.11)	1.02 (0.91-1.14)	1.06 (0.97-1.15)
4^th^ Quartile	1.11 (0.92-1.34)	0.93 (0.76-1.14)	0.99 (0.86-1.15)	1.10 (1.02-1.20)*

1^st^ Quartile is the reference. Models adjusted for maternal characteristics (age, race, and education) and state-level demographic factors.

In this large, national study, we found that residence in a state with a higher level of negative racial sentiment on Twitter was associated with an increased prevalence of maternal hypertension, particularly prepregnancy hypertension among racially minoritized groups. This association was markedly stronger during the period of heightened racial tension in 2020–2021. Our findings provide evidence that negative racial sentiment contributes to maternal health disparities in prepregnancy and gestational hypertension among racially minoritized groups and may be heightened by distressing external events.

Notably, in states with the highest levels of negative racial minority sentiment, prepregnancy and gestational hypertension were higher among all populations during the pandemic – with higher magnitudes of association for prepregnancy hypertension. However, patterns for gestational hypertension were not consistent across time and when examining race-specific negative sentiment. This observation could be partially attributed to the differing time frames between the two conditions; gestational hypertension develops over a shorter period usually during the second half of pregnancy, while prepregnancy hypertension encompasses a longer time period and is more closely tied to long-term factors and experiences, which is aligned with a life course perspective.

Throughout the study period, negative racial sentiment on Twitter toward Black individuals persisted and remained higher than negative racial sentiment toward Latinx and Asian individuals, and the association between high negative Black sentiment and increased prevalence of prepregnancy hypertension among this group persisted over time. There was a brief dip in negative racial sentiment on Twitter toward Black individuals when the Black Lives Matter movement gained momentum in the summer of 2020. However, this time period was also marked by race-related stressors from counter-movements, such as All Lives Matter (ALM), White Lives Matter (WLM), and Blue Lives Matter (Blue LM), which were associated with higher levels of implicit racism against Black individuals and subsequent rates of obesity [47]. From 2020−2021, the prevalence of prepregnancy hypertension was higher (19%) among Asian individuals in states with the highest level of negative sentiment toward Asian individuals, compared to states with the lowest levels of these sentiments. Rising anti-Asian discrimination during the COVID-19 pandemic, including discriminatory rhetoric like “Chinese virus” and “Kung Flu” [30,62,63], has been associated with increased stress, hate crimes, and negative health outcomes for Asian populations [27,30,45,64,65]. The peaks of negative racial sentiment in tweets during March of 2020 and 2021 align with the beginning of the COVID-19 pandemic and the Atlanta Spa shootings, which were periods of increased anti-Asian discourse and discrimination [27,30,64]. Collectively, these findings suggest that rising racial hostility toward Black and Asian communities during these two years could have contributed to increased prepregnancy hypertension among these groups. The overall societal stress response to heightened racial animus during this period may also explain why the prevalence of prepregnancy hypertension was higher among White individuals in states with the highest level of negative sentiment toward minoritized groups, compared to states with the lowest levels.

Our findings are consistent with and build upon previous research that explored trends in negative sentiment toward minoritized groups on social media platforms and the association between negative racial sentiment and adverse health outcomes among racial/ethnic minorities. Findings by Nguyen and colleagues have shown negative racial sentiment toward minoritized groups is associated with adverse birth outcomes, including LBW, VLBW, and PTB [10,11,26]. Given prior research showing maternal hypertension, particularly prepregnancy hypertension, is a large contributor to Black-white disparities in PTB [12], our study may explain one potential pathway through which negative racial sentiment increases adverse birth outcomes among racially minoritized groups – maternal hypertension. A major strength of our study is utilizing Twitter data to measure area-level negative racial sentiment, which allows us to explore environmental contexts of and exposures to cultural racism. The relative anonymity that online spaces and social media platforms provide may also encourage individuals to express opinions they may not share in person [66]. Through this novel approach, we can examine changes in the association between negative racial sentiment and health disparities over time and place, including in the wake of race-related events, like the Black Lives Matter movement and anti-Asian discrimination during the COVID-19 pandemic [27,28,30,64].

While this method is innovative, it is not without limitations. To be comparable to our previous work [10,11,27,28], we utilized the same sentiment model, and the keyword list is not exhaustive of all keywords that could reference racially minoritized groups. Tweets referencing different racial groups were analyzed for sentiment or emotional tone, which is distinct from expressions of racial prejudice. However, prior work has demonstrated that this measure is associated with implicit and explicit racial bias [25], as well as elevated risk for preterm birth and low birthweight among racially minoritized mothers [11]. The birth certificate data file is nationally representative and encompasses 99% of all births [67], and while hypertension has been shown to be underreported on state birth certificates, it is measured consistently over time [68], but the specific type of gestational hypertension disorder (e.g., preeclampsia) and the time of prepregnancy hypertension diagnosis are not assessed. While prepregnancy hypertension could have developed before the sentiment measure from Twitter and study period (i.e., prior to 2016), we observed temporal trends in negative racial sentiment and that its associations with prepregnancy hypertension aligned with periods of heightened racial discrimination. We also controlled for individual and state-level factors; however, there may be unmeasured confounders, which could explain these findings. Our E-value assessment suggests our observed findings could be susceptible to modest, unmeasured confounding [61]. However, the E-value alone cannot infer or refute causality, rather careful consideration should be made with respect to study design, sample size, and biologic plausibility that may explain a statistically significant association between negative area-level racial sentiment and prepregnancy hypertension among racially minoritized groups. Additionally, only 1 in 4 US adults uses Twitter [50], and Twitter users’ sociodemographics differ from the general population; they are younger, more highly educated, wealthier, and more likely to be from minority backgrounds [69,70]. Therefore, our findings may not be generalizable to the general population. Collectively, these limitations could have influenced our observed findings.

## Conclusions

Our study provides new insights into the impact of cultural racism on maternal health by integrating multiple years of nationally representative birth certificate data with Twitter data to examine the association between state-level negative racial sentiment and two types of maternal hypertension among multiple racial/ethnic groups. Our findings underscore the impact of societal-level racial sentiment on maternal health disparities and highlight the need for policies that address both overt racism and more subtle forms of racial sentiment, including online discourse. Further research is needed to examine how early-life and prepregnancy exposures to racism and discrimination, particularly during periods of heightened racial tension, affect maternal and child health inequities across the lifecourse. Findings from such research will provide additional insight into the long-term effects of racism and discrimination on maternal and child health inequities, and they may inform policies and interventions to reduce health inequities across racially minoritized groups. Public health and policy efforts to improve maternal health equity must extend beyond the clinic to address the pervasive and harmful effects of cultural racism. Policy efforts to combat online hate speech and promote racial justice may have direct and measurable benefits for the physical health of pregnant people and their infants [71–73].

## Supporting information

S1 Table Associations using prevalence rate ratios (PRRs) between state-level negative racial sentiment toward minoritized groups and hypertension type by pregnant individual’s race (all, racially minoritized groups, White) from 2016–2021, stratified by parity. (DOCX)

S2 Table Sensitivity analyses for associations using prevalence rate ratios (PRRs) between state-level negative racial sentiment toward minoritized groups and hypertension type by pregnant individual’s race (all, racially minoritized groups, White) from 2016–2021, adjusted for final selection of covariates as well as BMI, prenatal care initiation trimester, smoking during pregnancy, and complete smoking history. (DOCX)

S3 Table E-values for sensitivity analysis of unmeasured confounding in significant, adjusted associations using prevalence rate ratios (PRRs) between state-level negative racial sentiment toward minoritized groups and hypertension type by pregnant individual’s race. (DOCX)
