# Supplementary material for: The association between state-level negative racial sentiment and maternal hypertension in the US from 2016 to 2021: An observational study using Twitter data
Source: PLoS One. 2026 Apr 29;21(4):e0346564. doi: 10.1371/journal.pone.0346564 (PMC13127946; doi:10.1371/journal.pone.0346564)
Supplement: S1 Table — (DOCX) [file pone.0346564.s001.docx]

| **Supplemental Table 1. Associations using prevalence rate ratios (PRRs) between state-level negative racial sentiment toward minoritized groups and hypertension type by pregnant individual’s race (all, racially minoritized groups, White) from 2016-2021, stratified by parity** | | | |
| --- | --- | --- | --- |
|  | All  Adjusted PRR (95% CI) | Racially Minoritized Groups  Adjusted PRR (95% CI) | White  Adjusted PRR (95% CI) |
|  | First-born | | |
|  | N=8,324,788 | N=3,841,845 | N=4,482.943 |
| **Prepregnancy** |  |  |  |
| 2^nd^ Quartile | 1.10 (0.98-1.27) | 1.23 (1.03-1.46) | 1.04 (0.91-1.19) |
| 3^rd^ Quartile | 1.15 (0.97-1.36) | 1.19 (0.97-1.47) | 1.13 (0.97-1.32) |
| 4^th^ Quartile | 1.25 (0.96-1.64) | 1.35 (1.02-1.79) | 1.21 (0.93-1.57) |
| **Gestational** |  |  |  |
| 2^nd^ Quartile | 1.08 (0.94-1.23) | 1.15 (0.98-1.34) | 1.03 (0.91-1.17) |
| 3^rd^ Quartile | 1.11 (0.96-1.30) | 1.18 (0.99-1.41) | 1.08 (0.91-1.57) |
| 4^th^ Quartile | 1.17 (0.99-1.37) | 1.21 (1.00-1.46) | 1.14 (0.96-1.35) |
|  | Second-born or higher | | |
|  | N=13,129,547 | N=6,489,014 | N=6,650,533 |
| **Prepregnancy** |  |  |  |
| 2^nd^ Quartile | 1.13 (0.98-1.31) | 1.28 (1.06-1.54) | 1.02 (0.90-1.16) |
| 3^rd^ Quartile | 1.18 (0.98-1.42) | 1.27 (1.02-1.59) | 1.12 (0.95-1.31) |
| 4^th^ Quartile | 1.27 (0.97-1.67) | 1.38 (1.01-1.88) | 1.20 (0.92-1.56) |
| **Gestational** |  |  |  |
| 2^nd^ Quartile | 1.11 (0.98-1.25) | 1.19 (1.03-1.39) | 1.06 (0.95-1.17) |
| 3^rd^ Quartile | 1.12 (0.97-1.28) | 1.19 (1.01-1.40) | 1.09 (0.94-1.25) |
| 4^th^ Quartile | 1.20 (1.01-1.42) | 1.24 (1.02-1.50) | 1.18 (0.97-1.42) |
| 1^st^ Quartile is the reference. Models adjusted for maternal characteristics (age, race, and education) and state-level demographic factors. | | | |
