# Supplementary material for: The association between state-level negative racial sentiment and maternal hypertension in the US from 2016 to 2021: An observational study using Twitter data
Source: PLoS One. 2026 Apr 29;21(4):e0346564. doi: 10.1371/journal.pone.0346564 (PMC13127946; doi:10.1371/journal.pone.0346564)
Supplement: S2 Table — (DOCX) [file pone.0346564.s002.docx]

| **Supplemental Table 2. Sensitivity analyses for associations using prevalence rate ratios (PRRs) between state-level negative racial sentiment toward minoritized groups and hypertension type by pregnant individual’s race (all, racially minoritized groups, White) from 2016-2021, adjusted for final selection of covariates as well as BMI, prenatal care initiation trimester, smoking during pregnancy, and complete smoking history** | | | |
| --- | --- | --- | --- |
|  | All  Adjusted PRR (95% CI) | Racially Minoritized Groups  Adjusted PRR (95% CI) | White  Adjusted PRR (95% CI) |
|  | BMI Added to Model | | |
|  | N=21,057,903 | N=10,086,827 | N=10,971,076 |
| **Prepregnancy** |  |  |  |
| 2^nd^ Quartile | 1.13 (0.99-1.29) | 1.27 (1.06-1.52) | 1.02 (0.92-1.13) |
| 3^rd^ Quartile | 1.14 (0.96-1.36) | 1.24 (0.99-1.55) | 1.07 (0.93-1.24) |
| 4^th^ Quartile | 1.25 (0.94-1.64) | 1.36 (0.99-1.86) | 1.17 (0.90-1.51) |
| **Gestational** |  |  |  |
| 2^nd^ Quartile | 1.09 (0.97-1.23) | 1.17 (1.01-1.36) | 1.04 (0.94-1.14) |
| 3^rd^ Quartile | 1.09 (0.96-1.24) | 1.17 (0.99-1.38) | 1.04 (0.92-1.17) |
| 4^th^ Quartile | 1.16 (1.00-1.35) | 1.20 (0.98-1.46) | 1.13 (0.97-1.32) |
|  | Prenatal Care Initiation Trimester Added to Model | | |
|  | N=21,064,627 | N=10,075,044 | N=10,949,583 |
| **Prepregnancy** |  |  |  |
| 2^nd^ Quartile | 1.13 (0.98-1.30) | 1.27 (1.06-1.53) | 1.03 (0.91-1.17) |
| 3^rd^ Quartile | 1.18 (0.98-1.41) | 1.26 (1.01-1.57) | 1.13 (0.96-1.31) |
| 4^th^ Quartile | 1.28 (0.97-1.68) | 1.39 (1.02-1.88) | 1.21 (0.93-1.57) |
| **Gestational** |  |  |  |
| 2^nd^ Quartile | 1.09 (0.97-1.23) | 1.17 (1.01-1.35) | 1.04 (0.94-1.15) |
| 3^rd^ Quartile | 1.11 (0.97-1.27) | 1.17 (1.00-1.37) | 1.08 (0.94-1.23) |
| 4^th^ Quartile | 1.17 (1.00-1.37) | 1.20 (1.00-1.45) | 1.15 (0.98-1.36) |
|  | Smoking During Pregnancy Added to Model | | |
|  | N=21,430,959 | N=10,310,192 | N=11,120,767 |
| **Prepregnancy** |  |  |  |
| 2^nd^ Quartile | 1.12 (0.97-1.28) | 1.25 (1.05-1.50) | 1.02 (0.91-1.16) |
| 3^rd^ Quartile | 1.15 (0.97-1.37) | 1.23 (0.99-1.53) | 1.11 (0.96-1.29) |
| 4^th^ Quartile | 1.25 (0.96-1.63) | 1.35 (1.00-1.81) | 1.19 (0.92-1.54) |
| **Gestational** |  |  |  |
| 2^nd^ Quartile | 1.09 (0.96-1.22) | 1.16 (1.00-1.35) | 1.04 (0.94-1.15) |
| 3^rd^ Quartile | 1.10 (0.96-1.22) | 1.17 (0.99-1.37) | 1.07 (0.94-1.22) |
| 4^th^ Quartile | 1.17 (1.00-1.36) | 1.20 (0.99-1.45) | 1.15 (0.97-1.35) |
|  | Complete Smoking History Added to Model | | |
|  | N=21,426,929 | N=10,308,590 | N=11,118,339 |
| **Prepregnancy** |  |  |  |
| 2^nd^ Quartile | 1.12 (0.97-1.28) | 1.25 (1.05-1.50) | 1.02 (0.91-1.16) |
| 3^rd^ Quartile | 1.15 (0.97-1.37) | 1.23 (0.99-1.53) | 1.11 (0.96-1.29) |
| 4^th^ Quartile | 1.25 (0.96-1.63) | 1.35 (1.00-1.81) | 1.19 (0.92-1.54) |
| **Gestational** |  |  |  |
| 2^nd^ Quartile | 1.08 (0.96-1.22) | 1.16 (1.00-1.35) | 1.04 (0.94-1.15) |
| 3^rd^ Quartile | 1.10 (0.96-1.26) | 1.17 (0.99-1.37) | 1.07 (0.94-1.22) |
| 4^th^ Quartile | 1.17 (1.00-1.36) | 1.20 (0.99-1.45) | 1.15 (0.97-1.35) |
| 1^st^ Quartile is the reference. Main model adjusted for maternal characteristics (age, race, and education) and state-level demographic factors. | | | |
