# Supplementary material for: The association between state-level negative racial sentiment and maternal hypertension in the US from 2016 to 2021: An observational study using Twitter data
Source: PLoS One. 2026 Apr 29;21(4):e0346564. doi: 10.1371/journal.pone.0346564 (PMC13127946; doi:10.1371/journal.pone.0346564)
Supplement: S3 Table — (DOCX) [file pone.0346564.s003.docx]

| **Supplemental Table 3. E-values for sensitivity analysis of unmeasured confounding in significant, adjusted associations using prevalence rate ratios (PRRs) between state-level negative racial sentiment toward minoritized groups and hypertension type by pregnant individual’s race** | | | |
| --- | --- | --- | --- |
|  | **Observed Effect** | **E-value** | |
|  | Adjusted PRR (95% CI) | Point Estimate | Lower CI Limit |
| **2016-2021: Prepregnancy hypertension among racially minoritized groups** |  |  |  |
| 2^nd^ Quartile | 1.26 (1.05-1.51) | 1.83 | 1.28 |
| 4^th^ Quartile | 1.36 (1.01-1.83) | 2.06 | 1.11 |
| **2016-2019: Prepregnancy hypertension among racially minoritized groups** |  |  |  |
| 2^nd^ Quartile | 1.22 (1.06-1.41) | 1.74 | 1.31 |
| **2020-2021: Prepregnancy hypertension among all groups** |  |  |  |
| 4^th^ Quartile | 1.39 (1.06-1.81) | 2.13 | 1.31 |
| **2020-2021: Prepregnancy hypertension among racially minoritized groups** |  |  |  |
| 2^nd^ Quartile | 1.30 (1.04-1.63) | 1.92 | 1.24 |
| 3^rd^ Quartile | 1.29 (1.02-1.62) | 1.90 | 1.16 |
| 4^th^ Quartile | 1.51 (1.12-2.03) | 2.39 | 1.49 |
| **2020-2021: Gestational hypertension among racially minoritized groups** |  |  |  |
| 2^nd^ Quartile | 1.15 (1.10-1.31) | 1.57 | 1.43 |
| **2020-2021: Prepregnancy hypertension among White** |  |  |  |
| 4^th^ Quartile | 1.32 (1.03-1.69) | 1.97 | 1.21 |
| **2016-2021: Prepregnancy hypertension among Black** |  |  |  |
| 2^nd^ Quartile | 1.26 (1.08-1.46) | 1.83 | 1.37 |
| 4^th^ Quartile | 1.39 (1.05-1.84) | 2.13 | 1.28 |
| **2016-2021: Prepregnancy hypertension among Asian** |  |  |  |
| 4^th^ Quartile | 1.14 (1.01-1.28) | 1.54 | 1.11 |
| **2016-2019: Prepregnancy hypertension among Black** |  |  |  |
| 2^nd^ Quartile | 1.26 (1.04-1.53) | 1.83 | 1.24 |
| **2020-2021: Prepregnancy hypertension among Black** |  |  |  |
| 4^th^ Quartile | 1.31 (1.02-1.69) | 1.95 | 1.16 |
| **2020-2021: Gestational hypertension among White** |  |  |  |
| 4^th^ Quartile | 1.10 (1.02-1.20) | 1.43 | 1.16 |
| 1^st^ Quartile is the reference. Models adjusted for maternal characteristics (age, race, and education) and state-level demographic factors. | | | |
